# Use of Cytology in the Diagnosis of Basal Cell Carcinoma Subtypes

**DOI:** 10.3390/jcm9030612

**Published:** 2020-02-25

**Authors:** Paola Pasquali, Gonzalo Segurado-Miravalles, Mar Castillo, Ángeles Fortuño, Susana Puig, Salvador González

**Affiliations:** 1 Dermatology Department, Pius Hospital de Valls, 43800 Tarragona, Spain; 2 Dermatology Department, Hospital Ramón y Cajal, 28034 Madrid, Spain; gonzalosegmi@hotmail.com; 3 Dermatology Department, Hospital Clinic de Barcelona, IDIBAPS, University of Barcelona, Spain & CIBERER Barcelona, 08036 Barcelona, Spain; marcastillofort@gmail.com (M.C.); susipuig@gmail.com (S.P.); 4 Eldine Patología, 43006 Tarragona, Spain; afortunyo@eldinepatologia.org; 5 Medicine and Medical Specialties Department, Alcalá University, 28801 Madrid, Spain; salvagonrod@gmail.com

**Keywords:** skin cancer, diagnosis, cytology, basal cell carcinoma

## Abstract

Background: Basal cell carcinoma (BCC) is the most common skin cancer in the white population. Nonsurgical treatments are first-line alternatives in superficial BCC (sBCC); therefore, differentiating between sBCC and non-sBCC is of major relevance for the clinician. Scraping cytology possesses several advantages, such as an earlier diagnosis and scarring absence, in comparison to a biopsy. Nevertheless, previous studies reported difficulties in differentiating the different BCC subtypes. The objective of this study was to determine the capability and accuracy of scraping cytology to differentiate between sBCC and non-sBCC. Methods: In this retrospective study, cytological samples of histologically confirmed BCC were examined. Select cytological features were correlated to BCC subtypes (sBCC or non-sBCC). Results: A total of 84 BCC samples were included (29 sBCC; 55 non-sBCC). An inverse correlation between the diagnosis of sBCC and the presence of mucin, dehiscence, and grade of atypia in the basal cells was observed. The presence of medium and large basal cell clusters correlated directly to a sBCC diagnosis. The presence of clear cells is strongly associated with sBCC. Therefore, Conclusion: Scraping cytology is reliable in differentiating sBCC from other BCC subtypes.

## 1. Introduction

Basal cell carcinoma (BCC) is the most common skin cancer in the white population [1]. This slow-growing, malignant epithelial skin tumor predominantly affects older people; however, epidemiological data point out an increasing incidence, particularly in the younger population [2].

Although there is no global consensus on the classification of BCC subtypes, one of the most accepted divides them in a nodulocystic, adenoid, micronodular, infiltrative, morpheaform (sclerosing), keratotic, metatypical (basosquamous), pigmented, superficial, and ulcerative BCC. Other unusual variants are pleomorphic (giant cell), clear cell, signet ring cell, granular, infundibulocystic, metaplastic, shadow cell, and keloidal BCC [3].

Current guidelines for BCC management recommend a different approach depending on the BCC subtype. Thus, nonsurgical treatments are considered first-line treatments for superficial BCC (sBCC), whereas surgical alternatives are usually the first choice for other subtypes [4]. Therefore, BCC subtyping—or, at least, differentiating sBCC from non-superficial BCC (nsBCC)—is crucial for the clinician in order to choose surgical or nonsurgical treatments.

Currently, several non-invasive techniques, such as dermoscopy, high frequency ultrasound, and reflectance confocal microscopy, are used to identify the BCC subtype and support the treatment decision. However, histopathology remains the gold standard for BCC subtyping [5,6,7,8]. A skin biopsy is usually the technique performed for this purpose. The biopsy requires sample processing and it usually leaves a scar that, if the BCC is susceptible of nonsurgical treatment, would preferably be avoided, especially in cosmetically sensitive areas, such as the face.

Cytology is a non-commonly used technique in dermatology, unlike other specialties, such as gynecology. It has several advantages, such as earlier diagnosis, the absence of scars [9,10] and stitches, the sparing of local anesthesia and suture material, and it also saves the patient a trip back to an outpatients’ minor procedures clinic to have the stitches removed [11].

Nevertheless, one of the most important drawbacks of exfoliative cytology so far is that previous studies reported that it is unable to differentiate the tumor subtypes [12], and others have only suggested its potential to determine the BCC subtype without proving it [13]. Scrape smears of BCC typically show many cohesive epithelial fragments composed of tightly-packed small cells with uniform, oval, dark nuclei. The nuclear chromatin is dense, but granular and evenly distributed; the nucleoli are small and indistinct. The cytoplasm is scanty and cyanophilic. The marginal palisading arrangement of tumor cells, stromal fragments, and mucin may be seen [9,14,15]. However, BCC subtyping—or, at least, differentiating sBCC from non-sBCC—would be of major relevance to the clinician in order to choose surgical or non-surgical management for BCC.

The objective of this study is to determine the capability and accuracy of cytology in differentiating sBCC from non-sBCC.

## 2. Material and Methods

A retrospective study was designed. Lesions with histological diagnosis of BCC and in which cytology had been previously done were included in the study. The lesions were collected between May 2016 and September 2016 from an outpatient clinic of the Pius Hospital of Valls and Eldine Laboratory (Tarragona, Spain). A previous informed consent was obtained from all patients.

Dermoscopy was performed prior the procedure to exclude coexisting lesions. The samples for cytology were obtained by a firm scraping of the lesion after first removing any surface crust. Usually, a scalpel blade was used. The tissue obtained was spread onto a glass slide and immediately fixed with fixation spray and stained using Papanicolaou’s technique. Coverslips were placed on the slides with a Dibutylphthalate Polystyrene Xylene (DPX) mounting medium, a synthetic non-aqueous mounting medium for microscopy, and they were permanently filed. Cytological examination was done with a Leica DM750 microscope (Leica Microsystems, Wetzlar, Germany), and the image was taken with a Leica ICC50 camera (Leica, Wetzlar, Germany).

The cytological features were grouped into three broad categories: basal cells, squamous cells, and other findings. The system of evaluation is detailed in Table 1.

In this retrospective study, all the cytology smears were assessed blindly by a pathologist with 27 years of experience in cytology, with no cross-reference to the clinical notes or the routine histopathological report.

The biopsies were taken either by a shave biopsy or an excision following local anesthesia. They were fixed in 4% formaldehyde, routinely processed, and embedded in paraffin. Sections were stained with hematoxylin and eosin. Histopathological classification was based on the previously described standard criteria for each subtype, and included superficial, nodulocystic, and infiltrative BCC. Histopathologically, in the nsBCC group, nodulocystic and infiltrative BCCs were included.

Demographic data, such as age, sex, and anatomic location of the lesions were also collected. 

Statistical analysis was performed using a XLSTAT statistical package (version 2.01.16684, 2015), considering each BCC as an independent event. The results were expressed as mean and standard deviation and frequencies. The outcome dichotomous variable was set to the definite histopathological diagnosis of a superficial type of BCC or a non-superficial type of BCC (including nodular and infiltrative types). All separated cytological variables were included in the analysis. On the one hand, the analysis of variance (ANOVA) and Chi-Square test were used to compare univariate associations of cytological features with a diagnosis of sBCC or non-sBCC. On the other hand, multivariate associations were assessed by using discriminant analysis (multiple logistic regression model), with the purpose of identifying independently significant cytological criteria to define each BCC subtype. All *p*-values cited are two-sided and *p*-values less than 0.05 were considered statistically significant. The accuracy of cytology sensitivity, specificity, positive predictive value, and negative predictive value of cytology for the sBCC diagnosis were calculated by comparing the cytological results with the histopathological findings. The results were arranged in a 2 × 2 contingency table.

## 3. Results

A total of 84 BCCs were included in the study from 45 patients (38 men, 84.4%, and 7 women, 15.6%). The age ranged from 52 to 96 (mean 76.5). The most common location was in the head and neck (*n* = 52), followed by the anterior and posterior thorax (*n* = 28), and extremities (*n* = 4). The major size of the tumors ranged from 3.77 mm to 13.00 mm (mean 8.35 mm, 95% CI = 7.7–9.0 mm).

The cytological findings relevant to the sBCC and nsBCC groups are summarized in Table 2.

The results of the subtype BCC cytodiagnosis (Table 3) allowed us to calculate the accuracy of this technique. The sensitivity and specificity for BCC differentiation was 96.55% and 100%, whereas the positive and negative predictive values were 100% and 98.21%, respectively.

A multiple-group discriminant analysis, which included 18 parameters, correctly classified 75% of original grouped cases into superficial or non-superficial BCCs.

### 3.1. Superficial BCC Features

Upon cytological evaluation, the most frequent criteria of sBCC were moderate cellularity of the basal cells (16/29, 55.17%) forming groups with an equal distribution as large, medium, and small clusters (33.8%, 34.3%, and 31.9%, respectively, proportions), the presence of basal cell sheets (18/29, 62.07%), mild grade of basal cell atypia (25/29, 86.21%), and the absence of dehiscence (26/29, 88.67%).

**Figure 1:**
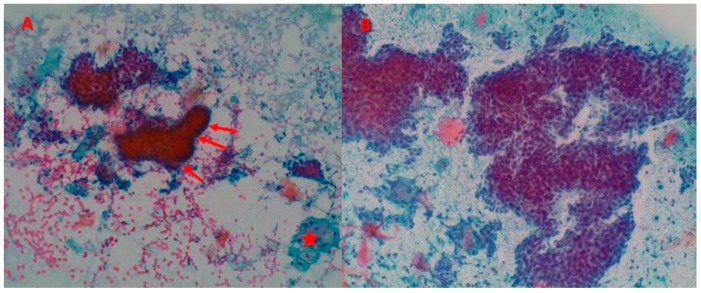
Basal cell cacinomas (BCCs). (**A**) Superficial basal cell carcinoma (sBCC): presence of two fragments composed of tightly packed small cells with mild atypia, peripheral palisade (red arrows), absence of dehiscence, and one cluster of clear sebaceous cells (red star) (Papanicolaou stain, ×100). (**B**) Non-superficial basal cell carcinoma (nsBCC): cellular smear with large clusters of basal cells with dehiscence and severe grade of atypia (Papanicolaou stain, ×200).

In regards to the squamous cells visualized in the cytological analysis, the predominant pattern was a moderate grade of squamous cellularity (12/29, 41.38%), with a high proportion of isolated cells (24/29, 82.76%). Other common findings were the presence of palisade cells (20/29, 68.97%) and clear sebaceous cells (13/29, 44.83%), the absence of mucin (27/29, 93.10%), and finally, more than 10 groups of stromal fragments (11/29, 37.93%) (Figure 1A).

As far as anatomical sites go, the most common location of sBCC was the trunk (22/29, 75.86%) lesions, and head and neck for the rest of the superficial tumors (7/29, 24.14%).

According to the results and the statistics applied, the analyses have shown a strong inverse correlation between the diagnosis of sBCC and the presence of mucin, dehiscence, and atypia in basal cells (the more severe the atypia, the less likely of it being a sBCC). Moreover, a significantly direct correlation between the presence of medium and large basal cell clusters has also been discovered on this BCC subtype.

### 3.2. Non-Superficial BCC Features

Upon cytological examination, the nsBCCs revealed high cellularity (41/55, 74.55%) and groups and sheets of basal cells (54/55, 98.18% and 33/55, 60.00%, respectively). According to the frequency of visualization, the order of the basal cell cluster size was large clusters (46.81%), followed by small (30.45%), and finally, medium (22.69%). In comparison to the superficial subtype, 34.55% (19/55) of nsBCCs showed the presence of dehiscence. Moreover, 10.91% (6/55) of the samples had basal cells with a severe grade of atypia, although a moderate grade was the most common (29/55, 52.73%) (Figure 1B).

**Figure 2:**
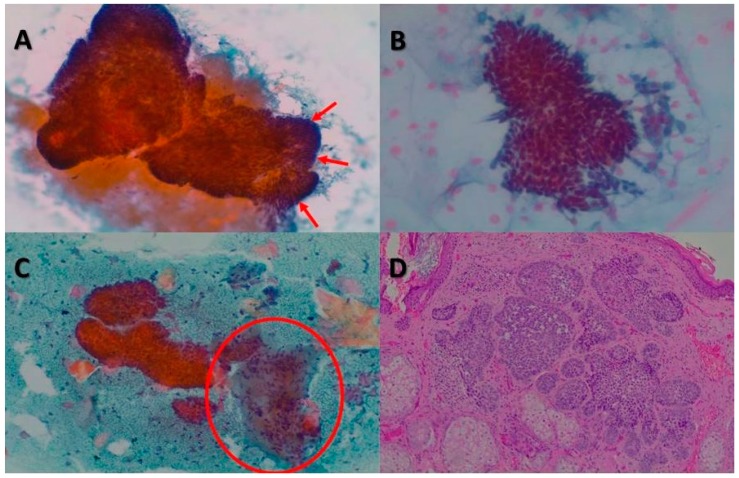
BCCs. (**A**) Cytology revealed large clusters of basal cells, mucin, and peripheral cells arranged in palisades (red arrows) (Papanicolaou stain, ×100). (**B**) Cytological image with a fragment composed of small basal cells with uniform oval, dark nuclei (Papanicolaou stain, ×200). (**C**) Group of basal cells accompanied by a stromal fragment (red circle) and keratinized squamous cells (Papanicolaou stain, ×100). (**D**) On histology, BCC showed a lobular pattern with islands and basaloid cells (hematoxylin and eosin, ×40).

Among the cytological features analyzed in the squamous cells, poor cellularity was the most common pattern found (18/55, 32.73%), followed by a total absence of such cells in 25.5% of nsBCCs. Finally, in 65.45% (36/55) of the samples in this group, a high number of isolated cells was reported. In contrast, the cytology did not show any clustered cells in 50.91% (28/55) of cases. Out of the last four items evaluated in the cytology, the palisade image was present in 56.36% (31/55) of the tumors. The results frequently showed the absence of mucin and clear cells (41/55, 74.55%, and 54/55, 98.18%, respectively). Finally, the most viewed stroma form was as <5 groups of stromal fragments (21/55, 38.18%) (Figure 2).

Most commonly, the nsBCCs developed on the head and neck (45/55, 81.82%); whereas their presence on the trunk and extremities was only 10.91% (6/55) and 7.27% (4/55), respectively. Regarding the subtypes, most nsBCCs were nodular (35/55, 63.63%), followed by infiltrative (14/55, 25.45%), and other subtypes (6/55, 10.90%).

## 4. Discussion

This study is unique in evaluating the reliability of the cutaneous tissue smear cytodiagnosis of sBCC, independent of the histopathological evaluation. We assessed the accuracy of the cytological criteria for differentiating sBCC from other subtypes.

The clinical and epidemiological characteristics of sBCC in our study were comparable with existing evidence. As previously reported, sBCC was most commonly located on the trunk, whereas nsBCCs developed more frequently on the head and neck area [16].

**Figure 3:**
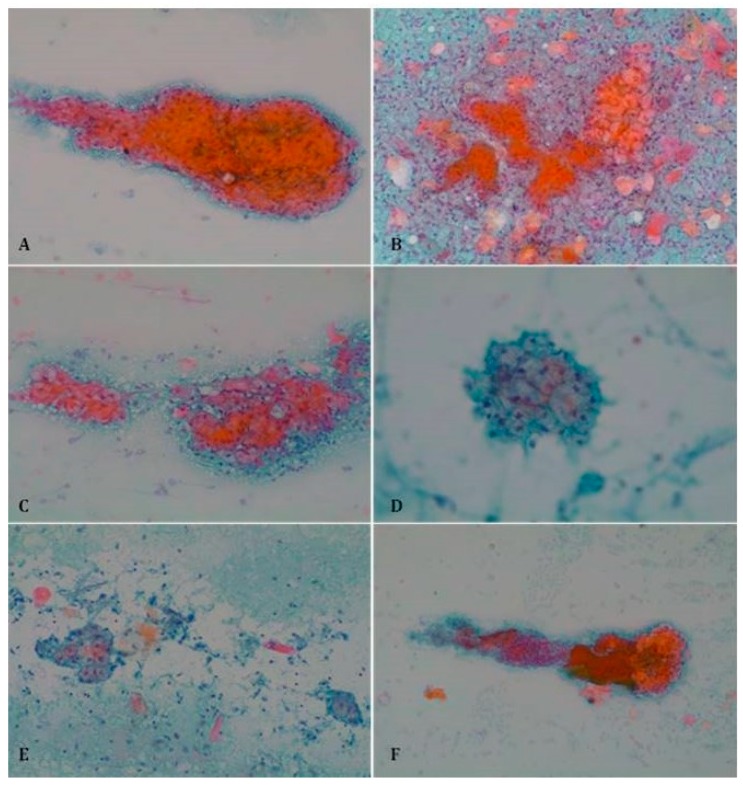
Clear sebaceous cells in sBCC (Papanicolaou stain). (**A**) Papanicolaou stain, ×100; (**B**) Papanicolaou stain, ×100; (**C**) Papanicolaou stain, ×100; (**D**) Papanicolaou stain, ×200; (**E**) Papanicolaou stain, ×100; (**F**) Papanicolaou stain, ×40.

Our results demonstrate that both classic and additional criteria may be present in superficial and non-superficial tumors, and accordingly, the presence of a single criterion cannot accurately predict the histopathological subtype. The clear cells represent an exception, being strongly associated with sBCC (Figure 3). In the only case where clear cells were found and nsBCC was established as the histopathological diagnosis, a biopsy also showed several focuses of sBCC. Furthermore, the frequent appearance of fragments containing a cluster of clear cells attached to a cluster of basal cells and stromal fragment could be explained by the superficiality of sBCC tumor proliferation and the intimate anatomical disposition of these three elements.

According to our results, the cytological pattern of sBCC is formed by the low cellularity of basal cells in contrast to a higher amount of squamous cells. Regarding the basal cells, they are characterized by mild atypia with an absence of dehiscence. In addition, the presence of a high quantity of stroma, clear cells, and peripheral palisade was shown to predict a sBCC subtype. In contrast, the detection of mucin could be suggestive of excluding the diagnosis of sBCC. All these findings may be explained by several reasons. On the one hand, low basal cellularity, mild-grade atypia, the absence of dehiscence, and peripheral palisade are related to the well-differentiation of the BCC. On the other hand, the high content of squamous cells and a high amount of stroma are associated with the superficiality of tumor proliferation.

We have shown that cytology could be a reliable diagnostic method for differentiating BCC between sBCC and nsBCC, with 98.81% of the lesions being correctly assessed. The only false negative reported was an ulcerative BCC instead of a sBCC with focal ulceration. It is common to establish an ulcerative BCC misdiagnosis, since this variant is usually accompanied by a lot of granulation tissue and inflammation. Although there are other techniques, such as reflectance confocal microscopy and optical coherence tomography, that may be used for the same purpose, their higher costs may limit the access to numerous dermatologists.

Limitations of the present study include the retrospective design that is subject to recall and observer biases. In addition, no investigator has previously noted the cytological variability of different subtypes of BCCs. During the analysis of the first samples, there were no cytological variables known as significant to differentiate each BCC subtype, and actually, it was not certainly known if any cytological criteria would be found. Moreover, since the pathologist assessed the cytology smears blindly with no knowledge of the clinical notes or the final histopathological diagnosis, an association of cytological patterns with BCC subtypes could not be made in advance. Moreover, the shaving biopsy may have missed some of the deepest BCC subtypes in the patients it was performed on [17,18]. Although dermoscopy was performed to exclude coexisting lesions, the location of most BCCs over photodamaged skin of the head and neck may hamper the recording of the squamous cell due to the background epidermal atypia [19].

Related to the technical procedures, there were some difficulties in subtyping BCC with cytodiagnosis. Mucin visualization was difficult in some cytological smears due to two reasons: one of them is explained by the presence of a blood background in the evaluation of the samples, because of the cytological technique itself (scraping); the second reason lies in the staining technique. While using Diff-Quick, the mucin is observed with an intense fuchsia tonality; in Papanicolaou’s staining, its intensity decreases significantly and the color is blue.

## 5. Conclusions

Cytological examination is easy to perform, does not require local anesthesia, saves time, is less expensive than a regular biopsy, and provides rapid diagnosis. Smear-taking for cytology is very well-tolerated, as it causes negligible trauma or discomfort to the patient.

Therefore, it can be performed (and, when necessary, repeated) even in apprehensive patients, and in sites where a biopsy has been proven to be difficult to obtain, or where aesthetic problems may arise, such as the face. 

Our study revealed significant differences in the cytological characteristics among superficial and other BCC subtypes, suggesting that a combination of this technique with other non-aggressive diagnostic modalities, such as a clinical examination, dermatoscopy, or ultrasound, may significantly enhance the preoperative subtype classification of the tumor. This is particularly relevant in clinical practice, where treatment modality is determined by the tumor subtype.

Additional studies are needed to investigate whether cytology could increase the accuracy of the preoperative subtype classification of BCC.

## Figures and Tables

**Table 1:** Cytological features of skin tissue scoring system definition.

Item		Definition		Score/Code
Basal cells	Cellularity	Total presence of basal cell clusters (plaques and/or groups) in the two extensions performed in each case (homogeneous behavior)	None	0
			Poor: <20	1
			Moderate: 20–100	2
			High: >100	3
	Groups	Three-dimensional cell clusters	Absence	0
			Presence	1
	Sheets	Two-dimensional cell clusters	Absence	0
			Presence	1
	Size	Size of cell clusters observed counted over 50 clusters in random fields. The percentages of the three groups add up 100%	Large clusters: >100 cells	0–100
			Medium clusters: 20–100 cells	0–100
			Small clusters: <20 cells	0–100
	Dehiscence	Quality of the peripheral cells of the cluster to be released from the primary cluster and remained isolated	Absence	0
			Presence	1
	Atypia	Abnormality in cells	Mild	1
			Moderate	2
			Severe	3
Squamous cells	Cellularity	Presence of squamous cellularity in the sample counted on 10HPF *	None	0
			Poor: <3	1
			Moderate: 4–6	2
			High: >6	3
Isolated cells		Quantity of dispersed single cells in the sample counted on 10HPF	0	0
			<3	1
			4–6	2
			>6	3
Clustered cells		Quantity of clustered cells in the sample counted on 10HPF	None	0
			<3	1
			4–6	2
			>6	3
Other findings	Palisade	Peripheral cells of the cluster disposed as palisade cells	Absence	0
			Presence	1
	Mucin		Absence	0
			Presence	1
	Stroma	Presence of stromal fragments (Fibrous, fibromyxoid, fibrovascular) loose or attached to other cell groups. Valued over the entire sample	None	0
			<5 groups	1
			6–10 groups	2
			>10 groups	3
	Clear cells	Presence of clear sebaceous cells	Absence	0
			Presence	1

* High power field.

**Table 2:** Cytological findings and distribution scores in superficial and non-superficial basal cell carcinoma (BCC).

Cytological Feature	Category	Superficial BCC (*n* = 29)		Non-Superficial BCC (*n* = 55)		*p* Value
		Mean (95% CI)	Frequency	Mean (95% CI)	Frequency	
Basal cells						
Cellularity	None	2.38 (2.09–2.67)	0%	2.64 (2.34–2.94)	0%	0.08
	Poor		3.4%		10.9%	
	Moderate		55.2%		14.5%	
	High		41.4%		74.6%	
Groups	Absence	1.00 (0.70–1.30)	0%	1.34 (1.04–1.64)	1.8%	0.02
	Presence		100%		98.2%	
Sheets	Absence	0.62 (0.40–0.84)	37.9%	0.60 (0.38–0.82)	40%	0.86
	Presence		62.1%		60%	
Size	Large clusters	33.8 (23.7–43.9)		46.8 (36.7–56.9)		0.01
	Medium clusters	34.3 (28.4–40.2)		22.7 (16.8–28.6)		0.01
	Small clusters	31.9 (22.3–41.5)		30.45 (20.8–40.1)		0.77
Dehiscence	Absence	0.10 (−0.09–0.29)	89.7%	0.34 (0.15–0.53)	65.5%	0.02
	Presence		11.3%		34.5%	
Atypia	Mild	1.14 (0.87–1.41)	86.2%	1.73 (1.46–2.00)	36.4%	<0.0001
	Moderate		13.8%		52.7%	
	Severe		0%		10.9%	
Squamous cells						
Cellularity	None	1.66 (1.17–2.15)	17.2%	1.38 (0.89–1.87)	25.5%	0.17
	Poor		20.7%		32.7%	
	Moderate		41.4%		20%	
	High		20.7%		21.8%	
Isolated cells	0	2.48 (1.88–3.08)	17.2%	2.02 (1.42–2.62)	29.1%	0.13
	<3		0%		5.5%	
	4–6		0%		0%	
	>6		82.8%		64.4%	
Clustered cells	None	1.52 (0.95–2.09)	34.5%	0.96 (0.39–1.53)	50.9%	0.04
	<3		13.8%		25.5%	
	4–6		17.2%		0%	
	>6		34.5%		23.6%	
Other findings						
Palisade	Absence	0.69 (0.46–0.92)	31%	0.56 (0.33–0.79)	43.6%	0.27
	Presence		69%		56.4%	
Mucin	Absence	0.07 (−0.11–0.25)	93.1%	0.25 (0.07–0.43)	74.5%	0.04
	Presence		6.9%		25.5%	
Stroma	None	1.62 (1.12–2.12)	27.6%	1.42 (0.92–1.92)	18.2%	0.37
	<5 groups		20.7%		38.2%	
	6–10 groups		13.8%		27.3%	
	>10 groups		37.9%		16.3%	
Clear cells	Absence	0.45 (0.30–0.60)	55.2%	0.02 (−0.13–0.17)	98.2%	<0.0001
	Presence		44.8%		1.8%	

**Table 3:** Evaluation of cytology in subtype BCC diagnosis.

	Histopathology	
Cytology	Superficial BCC	Non-Superficial BCC
Superficial BCC	29	0
Non-superficial BCC	1	55
